# Major Stressors and Coping Strategies of Internal Migrant Workers During the COVID-19 Pandemic: A Qualitative Exploration

**DOI:** 10.3389/fpsyg.2021.648334

**Published:** 2021-05-20

**Authors:** Akanksha Srivastava, Yogesh Kumar Arya, Shobhna Joshi, Tushar Singh, Harleen Kaur, Himanshu Chauhan, Abhinav Das

**Affiliations:** Department of Psychology, Banaras Hindu University, Varanasi, India

**Keywords:** catastrophe, COVID-19, stressors, trauma, migrants

## Abstract

COVID-19 forced lockdown in India, leading to the loss of job, crisis of food, and other financial catastrophes that led to the exodus migration of internal migrant workers, operating in the private sector, back to their homes. Unavailability of transport facilities led to an inflicted need to walk back to homes barefooted without lack of any other crucial resources on the way. The woeful state of internal migrant workers walking back, with all their stuff on their back, holding their children, was trending on social media worldwide. Their problem continued even after reaching home, including misbehavior of villagers, indifferent mannerism of family members toward them, inability to fulfill family responsibility, and financial crisis, which led to stress, fear, and trauma for these internal migrant workers. The present paper aimed to assess the idiosyncratic stressors of internal migrant workers throughout the pandemic era and their responses toward those problems, which helped them cope with it. In-depth semistructured telephonic interviews were conducted with 25 internal migrant workers who were working in different cities in India before lockdown. The analyses revealed that multiple stressors include financial crisis, unavailability of food, inability to continue education, inability to pay house rent, lack of support from neighbors and family, and other psychological stressors that affected them. However, they also tried multiple strategies to deal with the problems, including a cognitive appraisal of the problem and making oneself psychologically competent to deal with the situation. Social support of family and friends played a vital role in enhancing hardiness and increasing the level of happiness at scarce times. At the same time, some of them could not deal with stressors and opted for psychiatric help to manage the physical symptoms of stress.

## Introduction

The hazardous spread of COVID-19 in India in early 2020 led the government to impose an uncertain period of lockdown along with other rules including social distancing, use of mask, and quarantine. The interstate boundaries were sealed to get some relief from the spread of the pandemic. The rules were crucial to reduce the spread of the disease, but these affected all the strata of society adversely too. People who have saved resources had some privilege over those who have only limited resources. Then, there are people who do not have sufficient resources in their normal day-to-day life, and they have to struggle for their life on a daily basis. These people move away from their homes to earn their livelihood and run their families on normal days. They are also known as internal migrant workers who walk out of their homes to other places because of different reasons including oppression due to cultural reason, natural calamity, or due to infrastructural changes for the reason of better living and for overcoming poverty (Akinola et al., 2014).

Migrant workers constitute 37% of the whole Indian population, which is approximately 45.36 crore people according to the 2011 census of India. Migrant workers mainly include daily wage laborers working in the informal sector. They leave their villages due to the lack of agricultural land and other socioeconomic obstacles (Das, 2020). Although these workers make the foundation of Indian economy due to their large proportion of population relying on migration to metro cities to earn their livelihood (Suresh et al., 2020), they are one of the weakest sections of the social body (Yadav and Priya, 2020). They face greater number of problems in their daily life after migration than the native workers. Their problem ranges from prolonged poverty, improper residence, cultural differences, language difficulties at new place, lack of family proximity and support, discriminative behavior toward them because of their low socioeconomic status (Choudhari, 2020). The stressors affect them severely and hamper their mental health (Meyer et al., 2016). Due to least savings and the huge cultural differences at their place of work, they stay marginalized from the native groups and have a higher level of vulnerability for any threat than the native workers (Fasani and Mazza, 2020). Serious issues like pandemic, which also harms the resourceful person, have a double harmful impact on internal migrant workers due to the dual power of the pandemic crisis along with a poor working environment (Giorgi et al., 2020).

The lockdown imposed in India in March 2020 had shaken the life of migrant workers. These internal migrant workers, who were already facing a number of challenges, had to deal with a new set of challenges. They were living in small and overcrowded places but now had to follow the norms of social distancing, which is almost impossible (Guadagno, 2020). Loss of job and financial crisis started the new series of problems for them during the pandemic and had created a situation of scarcity, which further led to added stress, depression, and trauma. In such a chaotic condition when there was no money and job assurance to survive away from their home, a large number of people had to decide to go back to their homes without any proper transport facility, food unavailability on the way, and with a lot of fear and anxiety to reach their homes (Guadagno, 2020), which continued throughout the lockdown with a variety of other humiliations and discomfort. The whole situation of the pandemic affected the internal migrant workers harshly, but they tried to deal with it on their own level with different tactics. There are a few studies on the experiences of internal migrant workers during the pandemic; however, to understand the stressors beginning from the lockdown to the unlocking of the nation, and the ways they used to get rid of those stressors throughout the pandemic is not explored in depth in current literature. The present study was planned to understand the inconvenience Indian internal migrant workers faced during the global crisis of the pandemic. It explored a number of factors that acted as a challenge or threat in the life of internal migrant workers and affected their state of equilibrium. This study also uncovered the dynamic situation-specific approaches they used to deal with the stress for bringing a stage of mental balance.

### Rationale for the Present Research

At the time of the COVID-19 pandemic, the whole world faced a situation of scarcity in all the areas of life. All the activities were held at halt. People were facing health-related issues, psychological stress, and socioeconomic crisis. Millions of migrant workers lost their jobs due to the lockdown and were facing a catastrophe in their livelihood (Das, 2020). In such a situation, migrant workers were hit so hard that they were struggling for their survival and have to decide to go back to their homes barefooted (Lancet, 2020). Even after returning back home, these people faced multiple problems, which were persistent throughout the lockdown. Earlier studies focusing on coping have found that migrant workers strategize different techniques to reduce the level of stress. Religious practices, such as praying to God for help in reducing tension (Nakonz and Shik, 2009) at the time of crisis, were done. To deal with financial crisis, some of them opt for overtime jobs, doing more than one job, and employ more earners than one in the home (Datta et al., 2007). To deal with the social crisis of being alone, they make more friends and explore the new culture in the work place (Hack-Polay, 2012). In order to deal with social discrimination, people opt to either detach themselves from the native group and continue to be stigmatized in the new group or try to prove the stigmas as incorrect (Moroşanu and Fox, 2013). However, empirical studies focusing on the migrant workers, who are the major contributors of the urban and rural providence, their stressful situation, their ability to deal with those stressors, and their coping strategies in the light of COVID-19 pandemic have found little space in the present literature. The unique experiences of these people beginning from the loss of job to walking miles without resources to food scarcity to unusual behavior of family and villagers to going back to their work again are little known. The hardiness component, which held them strong despite the strenuous circumstances, remains unexplored in the present literature.

Some studies that were conducted on this population during the COVID-19 pandemic have shown that due to the novel coronavirus, there started a discrepancy in the growth of internal migrant workers (Das, 2020). Studies also focused on the level of vulnerability of migrant workers at the time of global pandemic and need for change at the political level (Fasani and Mazza, 2020), and a few of them assessed the metal health degradation of migrant workers at the time of COVID-19 (Choudhari, 2020). The studies also addressed the experiences of migrant workers at the epoch of the pandemic to suggest some changes in the context of policy making and implementation (Suresh et al., 2020). However, a lack of empirical investigation, to understand the stressors and the coping strategies of internal migrant workers and the role of family and previous circumstances of life in enhancing the level of hardiness in them, is found in the literature. The present study, therefore, explored the experiences of internal migrant workers and aimed to uncover the factors creating stress for them. The study also explored the ways in which they helped themselves to deal with the situation. The study also analyzed the whole episode from the beginning of the pandemic followed by loss of jobs to the time when people got back to the work investigating the whole range of emotions throughout the phase.

## Materials and Methods

For the purpose of the present study, qualitative approach has been utilized to explore and understand the individual experiences of migrant workers at the time of pandemic and to better understand the number of stressors influencing them and their ways to deal with the problems. Interpretive phenomenological analysis (IPA) was used to better understand the perspective of specific individuals in the context of the pandemic. It helped in presenting an account of lived experiences of migrant workers at the time of the novel coronavirus.

### Participants

A criterion-based purposive sample of 25 internal male migrant workers who were residents of the Varanasi district and were working outside their home district (both within as well as out of their home state) before the COVID-19 lockdown was recruited for this study. The age of the participants ranged between 21 and 41 years. The demographic details of the internal migrant workers are given in Table 1.

**TABLE 1:** Demographic characteristics of the study participants.

Variables	Variable levels	N (%)
Marital type	Married	16 (64)
	Unmarried	09 (36)
Family type	Nuclear	10 (40)
	Joint	15 (60)
Earning members	Only earning member	19 (76)
	One other earning member	06 (24)

The access to the study sample was gained with the help of a known contact who himself was an internal migrant worker. The person was contacted and requested to provide consent in the facilitation of the study. After the agreement, he was requested to provide the required information about other internal migrant workers who were working outside their home town for more than 6 months before the lockdown. After the generation of a list of people, they were contacted individually through a phone call and were requested to be a part of the present study after explaining the purpose of the study. Later, those who gave consent to provide their experiences were asked some questions to check if they come into the inclusion criteria. Later, an appropriate time slot was given according to their convenience for the interview.

### Inclusion and Exclusion Criteria for the Participants

The participants having the following characteristics were included in this study:

• The age of the person should be more than 18 years.
• The person should not be suffering from any physical and psychological illness.
• The salary of the person should range from 10,000 to 15,000 rupees.
• The person must be at the working place when the lockdown was imposed.

The participants must not have the following characteristics:

• The person of a female gender.
• The person not contributing significantly to the family income.
• The person working as migrant workers for less than 6 months.
• The person living with their family at the workplace.

### Procedure

The migrant workers were individually contacted and were explained the purpose, importance, and significance of the research work. They were than encouraged to ask anything they want to know about the interview and were ensured about the confidentiality and anonymity of their responses. They were also informed that they are free to answer only those questions only for which they felt comfortable and to refuse to answer the questions they do not want to. Informed consent with permission to record the audio of the conversation between the participant and the researcher was obtained before beginning the interview.

The semistructured interview was conducted at the time mutually agreed by the participants and the interviewer. The sequence of questions was not pre-decided, and the interviewer was free to decide the sequence of the question as per the flow of the interview. The interviewer was also free to ask some additional probe questions according to the demand of the situation. Some of the questions included in the present research work were, “For how long did you work outside your hometown?” “What was your thought when you first heard about the announcement of the lockdown?” “Why did you decide to go back to your home?” “What kind of difficulties did you feel after listening to that announcement?” “How did you come back to your home town?” “What were your fears on the way?” “What happened when you reached your village?” “What were your experiences in the quarantine?” “What were your thoughts throughout this strange situation?” “What were the physical and psychological problems you faced throughout this process?” “What did you do to deal with those problems?” “How did your family behave when you reached home?” “What are those things in the whole scenario from which you really felt stressed?” “What else did you do to decrease the level of stress throughout the lockdown?” “How are the things now?” “What type of change did you find in your work environment referring to pre-COVID and post-COVID environment?”

After the completion of the interview, the participants were thanked for their contribution in the research work. The interviews of all the participants were conducted in their native language. After completion of each interview, analytical memos were noted for better understanding of the non-verbal components and native meaning of words (specific for their culture). For most of the participants, the duration of interview ranged between 15 and 20 min.

## Analysis and Results

After collection of the data, transcription of the interview was done by the researchers. Furthermore, all the interviews were translated from Hindi to English, and later, proofreading was done by a native English speaker for the validation of the translation. After the completion of all the translations, the transcripts were then analyzed using the interpretive phenomenological approach (IPA) where we identified the experiences of the participants during migration, the stressors faced by them, and the coping strategies used by them to survive the situation. To analyze the data, the stepwise progression method was used. First, to get a deeper understanding of the experiences described by the participants, we read the transcriptions ample of times. We listened to the audio recordings, while we were reading the transcribed data to have a closer look at the data as much as possible, so that we leave no valuable finding untouched. In the next step, codes were assigned to each part of the interview. Then sorting of the data was completed to bring out themes from the assigned codes by bringing the similar components of each interview together and bifurcating the different components from it which helped in the formation of different themes out of the codes, which were elaborated later on.

In the next step, attempts were made to transform the transcriptions into a conceptual framework that was strongly connected to the original verbatim of the participants and to identify the emergent themes and subthemes, which were than clustered according to their similarities. In the following Table 2, major themes and subthemes identified in the analysis are illustrated.

**TABLE 2:** Major themes and subthemes that emerged from the interviews of migrant workers during lockdown due to COVID-19 pandemic.

Stressors	Description
1. Financial crisis • No transport facility • Food unavailability on the road while coming back home • Lack of food during lockdown • Money saved for education used for basic necessities • Inability to pay rent	It refers to the loss of money during pandemic due to the loss of job, which also leads to a number of other problems
2. Lack of social support • Lack of empathy by villagers • Family rejection	It refers to the unavailability of people who care and show concern to the person in need.
3. Psychological stressors • Uncertainty of: • Duration of lockdown • Future and job • Quarantine • Fear of spread of pandemic • Information from social media • Cases around self	It refers to the stressors that are consequences of the pandemic environment and results in the form of stress, anxiety, fear, and trauma
**Coping strategy**	
1. Problem-oriented strategy • Work and earn for a few days • Medication	It refers to the strategy aimed to deal with the problem directly to tackle the poverty during the pandemic and in dealing with physical symptoms of stress
2. Social support • Family’s support • Friends’ support • Owner’s support	It refers to the group providing emotional support by showing affection and thus strengthening the person’s ability to face the situation
3. Self-help thoughts • Self-motivation • Others in poorer situation • It is everyone’s problem • We are always in a problem	It refers to the cognitive processes, which help in overcoming problems by understanding the crisis better and finding their position under the present scenario

Table 2 illustrates major themes indicating the stressors faced by migrants during the lockdown and coping strategies used by them to survive the challenging situation. In the following sections, each of these themes and subthemes that were identified would be discussed by providing relevant excerpts from the experiences of the participants.

### Stressors

#### Financial Crisis

Almost each participant reported the challenge of managing finances during migration in the lockdown because of COVID-19. The issue of transport facility, food unavailability on the road while coming back home, lack of food during lockdown, money saved for education used for basic necessities, and inability to pay the rent turned out to be the finest financial stressors during their lockdown journey. No facility of transportation was available to participants, and thus, traveling and coming back to their hometown became a major stressor for them. For example, participant 9 reported:

*“We came barefooted madam. Sometimes, some vehicle gave us lift, sometimes truck gave lift and sometime bus. Those who take us on their vehicle drop us after sometimes as we did not have travel pass. Many more problems were there.”*

Similarly, participant 1 described the issue of food unavailability on the way while coming back home and that they had no money to buy food:

*“Whenever we used to stop for a break we knew we would get nothing to eat as we were out of money, so I thought it is better to keep on walking, and see whatever happens later, however, at a few spots in the journey there were some people offering food.”*

Participant 4 also described similar experiences:

*“What can we do now madam? If there was no hunger, there would have been no problems. Since the food was scarce, we used to eat one day and stayed hungry on the other day. We belong to a poor family madam, if we happened to have some land for farming, we could have to bear the situation. We have no livelihood source in the family; my father is no more, only a mother who has to be taken care off.”*

Similarly, participant 3 reported that the money saved for his education was used for arranging basic necessities:

*“I wanted to study; I had a desire for a good education. I was saving money for that but in this lockdown all money is spent on arranging food and necessities and now I am doing a job again to save enough money to take admission.”*

Similarly participant 11 reported his inability to pay rent:

*“We came back because the salary was slashed to 50% during lockdown, I have many responsibilities, I have a child of 2 years, and I was unable to pay my room rent with that half salary, so ultimately we had to decide to come back.”*

It is therefore evident that participants faced the challenge of managing the finances during migration due to the COVID-19 imposed lockdown, be it the issue of transport facility, unavailability of food on the road while coming back home, or the lack of food during lockdown, or the money they have saved for education was used for basic necessities and also their inability to pay rent. All these turned out to be the finest financial stressors during the migrant workers’ lockdown journey.

#### Lack of Social Support

Migrants who have no other option than to come back to their native places or hometown have faced the lack of social support, which also became a stressor for them during the lockdown and made their condition worse. Lack of empathy by villagers and family rejection were faced by some participants during and after their migration. For example, while describing the experience, participant 15 reported:

*“Villagers asked my family not to let us stay in our home and to not give food in utensils rather use leaves of banana trees. I used to eat on those leaves only. Later when villagers created a lot of trouble, I went into a nearby quarantine.”*

Similarly, while talking about the rejection by family, participant 7 reported that:

*“They said corona is widespread, since you are also coming from another place, stay out of home; they provided a “CHAARPAI” (Bed) to sleep and, asked to keep my belongings away*………… *They asked to clean my shoes and socks and gave me food in disposables utensils. I felt so bad. I thought I shouldn’t have come here*………. *no one was touching me, each one was just running away. Food was always kept on ground. Seeing such a situation I felt it is better for a person to die rather than witness such maltreatment.”*

It is therefore evident from the above examples that participants faced the lack of social support which became a stressor for them during the lockdown and made their condition worse. It can evidently be seen that lack of empathy by villagers and rejection by family were faced by the participants during their migration and even after that.

#### Psychological Stressors

Besides financial and social stressors, psychological stressor also prevail during the migration as there were uncertainty and fear of almost every other thing. The stressor was predominantly comprised of the uncertainty of the quarantine period, the uncertainty of the duration of lockdown, the uncertainty of jobs and the future, along with the fear of the spread of the pandemic. Either the participants were worried about cases around or were having information through social media made them more scared. Participant 23, for example, reported:

*“We were asked to report to the police station once we reached our home. We asked them why than they replied only for Corona’s testing you have to come. But they lied to us, they kept us in quarantine and we went unprepared*,………*. we had no extra clothes and no means to communicate with our family who kept on worrying about us, we didn’t know when this quarantine would end.”*

Similarly, participant 17 reported:

*“We didn’t know until when this lockdown will be there. Will it be for a week or will it be extended for months or more than that. We did not have permanent jobs to feel secured to get wages while sitting at home; we were always thinking about this lockdown period ending soon so that we could return to our work.”*

Similarly, participant 5 reported the uncertainty of jobs and the future:

*“We used to have tensions about almost everything, lost the job, how to manage the home, how I will provide food to my family members? From where would the eatables come? Job has become an endless worry, I felt I was depressed, there were only sleepless nights, only thoughts related to uncertainty of job come in the mind.”*

The participants could be seen reporting the fear about the spread of pandemic experiences during lockdown. They have discussed about the apprehension they had about cases of COVID 19 around them. They also described the fear they developed due to the spread of information over social media. For example, discussing about the fear of cases around self, participant 8 reported:

*“The camp in which we were staying one person was tested positive, and then I had to leave that camp as one by one it was spreading. I was too much affected by what was happening there. I just left that camp, I felt so nervous but by god’s grace nothing happened to me.”*

Similarly, participant 2 described the fear developed due to information through social media:

*“When our work stopped we just wanted to go back home. I was lying on my bed and saw news in my phone shared through social media that people are being buried live if found with corona. Knowing this my mind got disturbed. I immediately told my friend that I wanted to leave that place. And I just started walking from my place to my home. During the entire journey, if I found anyone coming closer I just got scared that if I am infected with Corona then I would also be buried live. During the whole journey I was fearful as I kept on thinking what would happen to my family?”*

From the above examples, it is evident that individuals have experienced the psychological stressors in terms of uncertainty and fear, which is reflected in their verbatim, and therefore, they were highly scared in terms of them and their family.

### Coping Strategy

#### Problem-Oriented Strategy

Despite facing several stressors during the COVID lockdown, almost each participant reported that they were also trying to cope with the challenging unwanted situations, by using problem-oriented strategy, i.e., to work and earn for a few days or by having medication. For example, participant 25 indicated how he used to work for a few days:

*“To have food at least for a few days we used to temporarily work at a few places.”*

Similarly, participant 1 reported that he could cope with medication:

*“Madam there was many tensions in my head, there was no work, no income and the responsibilities on my shoulders got piled up. I used to get puzzled as to how to manage all these. I got disturbed mentally. Due to the tensions, I got almost no sleep, for the same complaint I have to visit a psychiatrist. S/he told me that due to overthinking and ample tensions my condition has grown into depression, doctors gave me medication for 10 days and asked to visit again for the follow-up. Now I felt quite fine after taking those medicines.”*

It is therefore evident that even though each of the migrants faced ample stressors, they used problem-oriented strategy to cope up, and such strategy helped them as either they worked for a few days at the few places, or they went for medication if the problem continued.

#### Social Support

Participants also reported coping with the help of social support, i.e., either by support of family, friends, or with the support of their owner. When describing the support they got from family, participant 24 reported that:

*“I have two nephews; I used to pass my time playing with them. Used to talk to elder sister in laws they used to understand us and guide us.”*

Similarly, participant 6 received support from friends for coping with the challenges:

*“To reduce the apprehension I used to enjoy with friends. Meeting them, talking to them helped. I was satisfied with the fact that everyone is facing this challenge.”*

However, there are some participants who claimed that the support from their employer helped them in coping. For example, participant 9 reported that:

*“The owner of our work station gave us money for the needful and asked us to re-join when the work resumes.”*

From the above examples, it is evident that social support became a coping strategy for the workers during the period of the lockdown, and with the help of family, friends, and their work space owner, they became able to cope with the situation.

#### Self-Help Thoughts

One of the major ways to cope with any situation is to accept the challenge and change your thoughts. Doing the same, the migrants also used to cope by using self-help thoughts, by either motivating themselves, or by looking at others who are in a poorer situation, by bearing in mind that it is everyone’s problem, and also by understanding that they are always in a problem. For example, participant 14 reported coping through self-motivation:

*“Always felt to have patience and after some time everything would be fine, so we kept on doing that.”*

Similarly, participant 8 attempted coping through thinking of others in a poorer situation than him:

*“There were people who were coming by walking, some were coming via cycles. I felt that at least I have transportation, others are in more difficult situations, and I just kept taking precautions.”*

Similarly, there are a few migrant workers who thought that it is everyone’s problem, and this helped them cope. For example, participant 5 reported that:

*“We are not alone in this, there are people who are not having any work, so we think everyone is having problems and this would be fine soon.”*

However, there are a few participants, who coped by thinking and believing that we are always in a problem, like the verbatim of participant 17 who summarized that:

*“As we are poor we are used to having a challenge in life at any point of time.”*

It is therefore evident that self-help thoughts became a coping strategy for the individuals during the period of the lockdown, and by changing various thoughts and beliefs, they were able to fight enough with the challenging situation they were facing during lockdown.

## Discussion

The beginning of the global pandemic followed by the lockdown is proof of a global catastrophe in the life of billions of people, but it will be considered as a death blow in the life of internal migrant workers. Those people were already facing a number of problems, but the pandemic exposed them to new direct and indirect challenges. The present study was conducted on internal migrant workers who have to come back home due to the pandemic situation. To explore their unique experiences of stressors and coping strategies, they opted to reduce the impact of those stressors.

The results of the present study are in the line of previous works on internal migrant workers at the time of COVID-19 (Choudhari, 2020). Studies synchronize the situation of internal migrant workers due to sudden loss of jobs (Jha and Kumar, 2020), helplessness due to the unavailability of public transportation facilities (Campion et al., 2020), crisis of food during the lockdown (Gowda, 2020), and other major stressors (Fasani and Mazza, 2020) with the previous studies. However, the present study adds to the existing literature by addressing the ways with which they coped up with the emerging crisis including the problem-oriented coping strategy, emotion-oriented coping strategy, and cognitive appraisal of the problem. Internal migrant workers, while having a stressful situation, were trying on multiple ways to get rid of the strain physically as well as psychologically.

Financial crisis had always been the major issue for migrant workers (Akinola et al., 2014). Poverty has been the biggest fight for them. The situation of the pandemic increased the seriousness of the problem. Even before the lockdown, the income of workers was reduced creating hurdles in accessibility of basic necessities. Loss of job led to increased tension and stress of migrant workers (Campion et al., 2020).

Due to financial scarcity, migrant workers faced hunger (Sarmin, 2020). The inability to earn and eat forced migrant workers to return back to their native place. However, due to hunger, many of them lost their lives, and a few committed suicide (Bhagat et al., 2020). The hunger also affected the mental health of the people leading to depression. One other reason of returning back was the difficulty to pay the rent of the house at the work place imposing the decision of coming back home.

The implementation of the decision to return back home was not easy either. The migrant workers had to rely on public transportation for any traveling (Fasani and Mazza, 2020), which was blocked during the lockdown creating a situation of uncertainty for them. They wanted to reach their home in any way possible. Those who were walking back were also stressed. Traveling increased mental tension for workers due to the fear of spread of the coronavirus, which affected their mental health (Dam et al., 2020).

The loss of source of income increased the panic of workers, and they started to use their saved money. Many people used their saved money for survival during the lockdown. People who saved money to get a good education dropped their plans and used the savings for the fulfilment of basic amenities.

Due to financial scarcity, people wanted to come back to their villages to have some relief from financial problems as well as to have some emotional support, but the problem does not end even after reaching their villages. The stigmatization by neighbors that every person coming from other places is infected with the coronavirus and needs to stay away from the village even after being quarantined filled the internal migrant workers with agony and hatred toward people. Studies have shown that the pandemic and lockdown have led to many psychological and behavioral repercussions such as fear, worries, stigmatization and discrimination, xenophobia, prejudice, negative attitude toward immigrants, conflicts in intergroup relations, etc. (Ji et al., 2019; Ahorsu et al., 2020; Troisi, 2020). Social support is proven to be one of the most crucial components for good mental health (Wong and Leung, 2008), but is not available to them. Such social exclusion impairs the mental health and sometimes brings suicidal ideation in the mind of migrant workers.

The behavior of family members put greater impact and was unacceptable for migrant workers. The family acts as a primary support group. Studies reported that in normal times, due to cultural differences, internal migrant workers stay homesick for a longer period of time (Hack-Polay, 2012), and being with the family gives them strength. However, at the time of global pandemic, when people reached their homes, they were expecting warmth and proximity with the family, but the situation was incongruent from their expectations. Family members do not come close and are instructed to stay out of the home and eat on banana leaves rather than on plates, which hurt the workers seriously. Internal migrants reported feeling of detachment and remorse after such a response from their families.

Other than social stressors, internal migrant workers were suffering from psychological stresses too. The complete epoch of the pandemic had acted as a “period of uncertainty.” The level of ambiguity was way too high for internal migrant workers ranging from uncertainty of jobs, lockdown, food, sanitation to uncertainty of the process of quarantine (Smith and Wesselbaum, 2020). This uncertainty was followed by fear and trauma. Such fear of crisis leads to social dysfunction and other mental illnesses including tension, depression, and anxiety (Giorgi et al., 2015).

Internal migrant workers view different behavioral options to reduce these stresses. The response behavior helps in the reduction of tension and sometimes increases the capability of persons to deal with the situation. Such behaviors are known as coping strategies (Bargiel-Matusiewicz and Omar, 2016). Migrant workers sometimes aim to directly deal with the situation, while sometimes, they act on the management of thoughts (Folkman and Lazarus, 1988) to maintain emotional balance. They reported working for a few days even during the lockdown whenever they get a chance like sowing in agricultural farms, carrying loaded baggage, etc., to earn a livelihood and get their basic needs fulfilled, while those who cannot deal with the stress gets depressive physical symptoms including sleeplessness, loss of appetite, and psychomotor retardation due to which they have to rely on medicines.

The situation of the COVID-19 cannot be controlled by anyone; thus, people try to manage their emotions rather than the situation itself. Social support helped in diminishing the stressful situation and in controlling its physical symptoms (Turner, 1983; Cohen and Wills, 1985; Kessler and McLeod, 1985). At the workplace, when a person stays with a group and feel a sense of belongingness, it helps to deal with the challenges in the office and to get relief from burnout and, thus, helps in maintaining emotional equilibrium (Chen et al, 2020). The significant person, with a feeling of care and concern toward the affected stressed individual help in acting as an assistant for stress management (Thoits, 1986). Gariépy et al. (2016) found that social support plays a significant role in dealing with depression. The spouse plays a really important role in the adjustment of the person in the new situation of the lockdown, which is supported by previous research. Along with the workplace, family support helps in the lightening of emotional burden at other times of crisis as what happened in the lockdown. The family in the beginning phase became a stressor for internal migrant workers, but after few days of reassurance that the person is out of danger of any risk of infection with the life-threatening virus, the family members played a very important role in minimizing their stress. Family members, along with providing financial support, gave emotional support by making the person understand that everything is under control. A few people enjoyed a lot being in the family as they get very less time with them in normal days. The present research shows that internal migrant workers, living in a joint family, were less stressed and happier than people living in nuclear families after the specific time of relief from anxiety of infection. Friends also helped by providing financial support as well as emotional support, which facilitated in reducing tension. The family helps in the regulation of behavior, while friends and significant others help in uncovering the different options available for coping (Fondacaro and Moos, 1987).

Other than outside factors, the person can strengthen himself by cognitive appraisal of the situation and increase their ability to deal with the situation. The person tries to gather information about the stressor, analyze the situation to understand the options available to them to tackle the situation, and then choose one of the best possible options (Janis and Mann, 1977). The internal migrant workers tried to motivate themselves to be patient in the present moment and wait for the future during which, they will work hard to compensate for the present loss. Some of them chose social comparison of the problem as a tool to sympathize with the self by viewing others in a greater problem than one’s own. On the other hand, a few migrant workers viewed the universalization of the global pandemic to feel that everyone is with them in the problem and will come out of it for sure. Some explained their helplessness by telling that they always have some types of crisis, and this is nothing new for them.

## Conclusion

The findings of the present research shows that internal migrant workers were highly affected due to the global pandemic, which bombarded multiple stressors on them causing physical stress, emotional stress, and social stress in their lives, which were created due to financial crisis, lack of social support, and fear and uncertainty related to the global pandemic. However, the workers looked after the options available to them and chose problem-oriented strategy by directly dealing with the crisis, emotion-oriented strategy by family and friends’ support, as well as cognitive appraisal of the pandemic situation by bringing optimistic thoughts to deal with these stressors. The present work strengthens the earlier studies on uncovering the stresses of migrant workers along with suggesting available options to deal with them and emphasizes the role of family and proper emotional support in the management of pandemic-related stress among internal migrant workers.

### Limitations and Future Direction

The present study makes a significant addition to the existing literature on the threats faced by internal migrant workers at the time of COVID-19. It helped in understanding the one-to-one experiences of workers and in better analysis of their perception of the emerged situation not available in previous researches. It also helped in investigating a number of strategies that helped them in dealing with the risks of the scenario. However, there are a number of limitations in the present paper. The experiences of female internal migrant workers were not included in the study due to the situational constraints caused by the pandemic, which should be further assessed in future researches. Along with that, the number of participants was also less in this study, again due to the pandemic-imposed rule of staying at home and social distancing, which inhibits its generalizability. However, the nature of the qualitative work is not generalizability but the illustration of themes (Creswell, 2014), which is done in-depth for the present study making this paper unique in this type. Another work can also be done with the quantitative analysis with a larger sample to assess how the present themes generalize to the population of internal migrant workers.

## Data Availability Statement

The raw data supporting the conclusions of this article will be made available by the authors, without undue reservation.

## Ethics Statement

Ethical approval was not provided for this study on human participants because all procedures followed in this study were in accordance with the APA’s ethical standard and with the Helsinki declaration of 1964 and its later amendments. The patients/participants provided their informed consent to participate in this study.

## Author Contributions

YA, TS, and SJ conceptualized the study, developed interview protocols, reviewed the draft, and provided important feedback. AS, HK, HC, and AD collected and transcribed interviews. AS and HK analyzed the data, prepared initial codes, and wrote the result draft. AS wrote the first manuscript draft and prepared the final manuscript draft. All authors reviewed and approved the final manuscript draft for submission.

## Conflict of Interest

The authors declare that the research was conducted in the absence of any commercial or financial relationships that could be construed as a potential conflict of interest.
